# Proteomic analysis of four *Clostridium botulinum* strains identifies proteins that link biological responses to proteomic signatures

**DOI:** 10.1371/journal.pone.0205586

**Published:** 2018-10-15

**Authors:** Brooke L. Deatherage Kaiser, Karen K. Hill, Theresa J. Smith, Charles H. D. Williamson, Paul Keim, Jason W. Sahl, Karen L. Wahl

**Affiliations:** 1 Chemical and Biological Signature Science Group, Pacific Northwest National Laboratory, Richland, Washington, United States of America; 2 Bioscience Division, Los Alamos National Laboratory, Los Alamos, New Mexico, United States of America; 3 Pathogen and Microbiome Institute, Northern Arizona University, Flagstaff, Arizona, United States of America; Institut Pasteur, FRANCE

## Abstract

Microorganisms alter gene and protein expression in response to environmental conditions to adapt and survive. Whereas the genetic composition of a microbe represents an organism’s biological potential, the proteins expressed provide a functional readout of the organism’s response to the environment. Understanding protein expression patterns in response to specific environmental conditions furthers fundamental knowledge about a microbe, which can be especially useful for understudied organisms such as *Clostridium botulinum* examined herein. In addition, protein expression patterns that reproducibly occur in certain growth conditions hold potential in fields such as microbial forensics, in which determination of conditions in which an unknown possible biothreat sample had been grown may be important. To investigate the identity and reproducibility of protein profile patterns for varied strains, we defined the proteomic profiles of four Group I strains of *Clostridium botulinum*, a Category A biothreat agent and the organism responsible for the production of the botulinum neurotoxin (BoNT), in two different culture media grown for five days. The four *C*. *botulinum* strains produced one of three neurotoxins (BoNT/A, /B, or /F), and their protein profiles were compared to that of a fifth non-toxigenic strain of *C*. *sporogenes*. These strains each had DNA sequences available to assist in accurate protein identification. Differing culture growth phase, bacterial strain, and growth medium resulted in reproducible protein profiles, which were used to calculate relative protein abundance ratios as an internally normalized metric of microbial growth in varying conditions. The resulting protein profiles provide functional information about how four Group I *C*. *botulinum* strains and a *C*. *sporogenes* strain respond to the culture environment during growth and explores the feasibility of using these proteins to characterize unknown samples.

## Introduction

Genome analysis provides a significant amount of information about an organism’s biological potential, but complementary functional information, such as the proteome, provides a view of an organism’s reality; that is, its response to the environment in which it is growing. Understanding such responses provides predictive power, which could be leveraged for forensic, clinical, and environmental applications, or any application in which analysis potentially relies on a single sample [1]. For example, if it is known that an organism consistently expresses a set of proteins as part of a metabolic response to a growth medium, then observation of those proteins in an unknown sample could facilitate deduction of characteristics of the growth environment from which it came. Most importantly, this would not be a “blind signature” of unrelated proteins that may be expressed, but rather a view of functional output with foundations in the underlying organism biology.

*Clostridium botulinum* is a Gram-positive anaerobic spore-forming bacterium best known for the production of the botulinum neurotoxin (BoNT), which causes a flaccid paralysis known as botulism in humans and animals. Because botulism is a public health concern, much research has focused on the incidence, clinical diagnostics, therapeutics, toxin expression, and mode of action of the neurotoxin [2–5]. To date, a few transcriptomic studies involving *C*. *botulinum* gene expression have been performed using microarray and qtRT-PCR techniques [6, 7], but proteomic studies of *C*. *botulinum* have primarily focused on expression of the neurotoxin and the toxin cluster proteins. Thus, despite the importance of *C*. *botulinum*, few studies have been performed to date that examine the expression of all proteins within the organism. The present state of *C*. *botulinum* global proteomics research can be summarized by the following quote: “Compared to the transcriptome, the proteome of *C*. *botulinum* is an unknown abyss as BoNT has been the primary focus of the field to the detriment of the study of the other proteins produced. Without further studies, *C*. *botulinum* will continue to be undefined on the protein level and will be limited in its understanding of other clostridial species.” [8] Therefore, the goals of this study were two-fold: 1) to use mass spectrometry complemented by genetic sequence data to characterize the protein profiles of four strains of Group I *C*. *botulinum* that express either BoNT/A, /B, or /F, and non-toxigenic *C*. *sporogenes*, and 2) to explore whether there are protein expression features in common among multiple *Clostridium* strains that can be attributed to biological processes and might prove useful in the characterization of unknown samples.

BoNT-producing Clostridia are phylogenetically diverse and can be considered multiple species based on comparisons of its 16S rRNA sequences [9, 10]. The description of a strain of *C*. *botulinum* can include its Group designation, toxin type, toxin subtype, and also information about its cluster of toxin-associated genes. Seven distinct BoNTs (A-G) are recognized [11]. *C*. *botulinum* Group I strains produce BoNT/A, /B, or /F, and they may produce more than one toxin, such as BoNT/A and /B or BoNT/A and /F. The *bont* genes in different strains can also contain nucleotide and amino acid variation, creating genetic variants called subtypes, which are designated by a number after the toxin type such as *bont*/A1, /A2, /A3, or *bont*/B1, /B2, etc. [11]. Chimeric toxins are designated with double letters, such as CD. Some of these variants are the result of recombination events within the toxin gene, including the recently described toxin type HA that contains a toxin gene sequence similar to both *bont*/A and *bont*/F5 [12]. Finally, the toxin gene is expressed along with several other toxin cluster genes as part of a botulinum toxin complex. The toxin-associated genes are known as either the hemagglutinin (*ha+*) cluster or orfX (*orfX+*) cluster, and are associated with different toxin types: BoNT/A, /B, /C, /D and /G are associated with HA complex proteins, while BoNT/A, /E and /F are associated with one or more orfX proteins [13].

In 2007, the first genomic sequence of a Group I *C*. *botulinum* strain ATCC 3502 was published [14]. Now the availability of additional genomic sequences (and their predicted proteins) from multiple *C*. *botulinum* strains provides the opportunity to study its proteomics. This study examined the proteome of four toxigenic Group I *C*. *botulinum* strains that express either BoNT/A, /B, or /F. One non-toxigenic *C*. *sporogenes* strain was included for comparison. Strains were cultured in two different media, and cell mass and cell culture supernatant (SN) were collected at three time points for proteome analysis by mass spectrometry. Consistently expressed proteins integral to *Clostridium* metabolism and growth were identified in this, to our knowledge, first large shotgun proteomics exploration of *Clostridium* global protein expression.

## Materials and methods

### Bacterial strains and growth conditions

**Fig 1:**
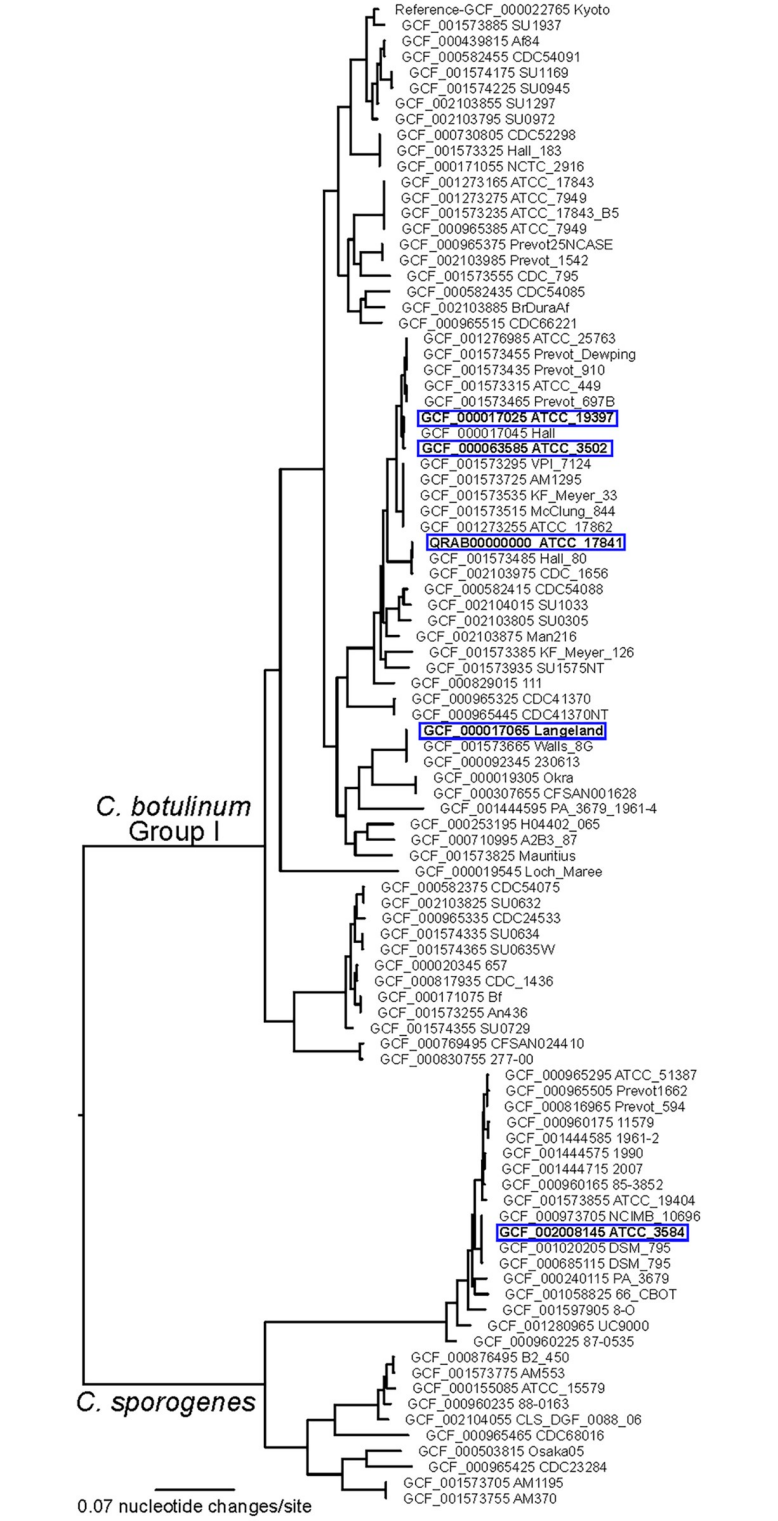
Phylogenetic tree of *C*. *botulinum* Group I and *C*. *sporogenes* Isolates. A maximum likelihood phylogeny inferred from core genome single nucleotide polymorphisms (SNPs) to show the strains analyzed in this study in the context of other *C*. *botulinum* Group I and *C*. *sporogenes* strains. Strains used in this study are highlighted in blue boxes.

Table 1 lists the five *Clostridium* strains that were analyzed in this study: *C*. *botulinum* ATCC 3502 Hall 174 (HA+, toxin type A1), *C*. *botulinum* ATCC 19397 NCTC 7272 (HA+, toxin type A1), *C*. *botulinum* ATCC 17841 McClung 1347 (HA+, toxin type B1), *C*. *botulinum* ATCC 35415 Langeland (orfX+, toxin type F1), and *C*. *sporogenes* ATCC 3584 (non-toxigenic). These four strains of *C*. *botulinum* and the *C*. *sporogenes* strain were selected because of the availability of genomic sequence data that could provide predicted protein information for the strains (Accession numbers noted in Table 1). In addition, the strains were all within *C*. *botulinum* Group I (which appear similar to each other using genomic sequence comparisons, described below and Fig 1), express any one of three toxins BoNT/A, BoNT/B, or BoNT/F, and represent the two toxin clusters (HA+ or orfX+).

**Table 1:** Strains used in this study.

Species	Strain	Toxin Type	Accession Number
*C*. *botulinum*	ATCC 3502 Hall 174	HA+ A1	NC_009495.1
*C*. *botulinum*	ATCC 19397 NCTC 7272	HA+ A1	NC_009697.1
*C*. *botulinum*	ATCC 17841 McClung 1347	HA+ B1	QRAB00000000
*C*. *botulinum*	ATCC 35415 Langeland	orfX+ F1	NC_009699.1
*C*. *sporogenes*	ATCC 3584	Non-toxigenic	MRAC00000000.1

Samples were generated by inoculating 100 mL TPGY broth (50.0 g/ L trypticase peptone, 5.0 g/ L Bacto peptone, 4.0 g/ L glucose, 20.0 g/L yeast extract and 1.0 g/L cysteine-HCL pH 7.4) or Brain Heart Infusion broth (BD BBL Brain Heart Infusion Modified and 1.0 g/L cysteine-HCL pH 7.4). These media were selected as they are commonly used for culturing *C*. *botulinum*. A single bacterial colony was used to inoculate an overnight culture of 100 ml TPGY. One ml of the culture at an OD_600_ of 1.0 (Thermospectronic spectrophotometer model 40001/4), was used to inoculate a 100 ml bottle of TPGY or BHI media. Cultures were incubated anaerobically at 30°C, and two 10ml samples were removed at three points: when the OD_600_ = 0.4, OD_600_ = 0.8, and at 5 days post-inoculation. These three time points were chosen to represent an early and late exponential, and stationary phase cultures, to determine if there were observable differences between these growth phases. The culture aliquots were autoclaved for 60 minutes, then frozen at -70°C prior to protein analysis. In this experimental design, each strain/medium combination was cultured twice on two separate days to generate biological replicates for protein analysis.

### Experimental biosafety precautions

The experiments were performed in two locations: the culturing of the *C*. *botulinum* and *C*. *sporogenes* was performed in an approved select agent (SA) laboratory, while the protein analysis was performed in BSL-1 mass spectrometry laboratory. The 10 ml aliquots for protein analysis were removed from the SA laboratory only after the 10 ml aliquots were autoclaved and tested for nonviability, as described below. In the SA laboratory all cultures were incubated in secondary containment within an incubator and all manipulations were performed under a certified biosafety cabinet (BSC). The OD readings were performed within the BSC using 1.0 ml disposable cuvettes with disposable caps for containment. At the appropriate OD, two 10 ml aliquots of the culture were pipetted into a conical Corning centrifuge tube and autoclaved for 60 minutes. After the autoclave cycle was complete, 5% of the total volume of each of the 10 ml autoclaved aliquots (500ul) was pipetted into100 ml TPGY or BHI media and incubated for 48 hours at 30°C in secondary containment. Two controls were always included in the nonviability testing. One control included 100 ml media that was not inoculated; the second control included inoculation of the media with a 500ul aliquot of a viable *C*. *botulinum* culture. Following 48 hours of incubation, nonviability of the 10 ml aliquots was determined if: 1) no bacterial growth was observed in the media inoculated with the autoclaved cultures, 2) no bacterial growth was observed in the uninoculated control medium, and 3) bacterial growth was observed in the control *C*. *botulinum* inoculated medium. Following the results of the nonviability testing, the samples could then be removed from the SA laboratory and stored at -70°C prior to protein analysis.

## Genome sequencing and phylogenetic analysis

The genome sequence of *C*. *botulinum* ATCC 17841 was determined as part of this study. DNA was extracted with a DNeasy kit (Qiagen, Valencia, CA, USA). Whole genome sequencing was conducted on the Illumina MiSeq platform at TGen North (Flagstaff, AZ). The genome was assembled with the SPAdes assembler v3.11.1 [15] and was annotated with Prokka v1.13 [16]. The 5 strains analyzed in this study were put into a broader phylogenetic context using core genome single nucleotide polymorphism (SNP) analysis similar to [17]. Genome assemblies were aligned to a reference assembly (GCF_000022765.1—*C*. *botulinum* Kyoto-F) with NUCmer [18, 19], and SNPs were called with NASP [20]. Duplicated regions of the reference were identified with NUCmer self-alignments and removed from the analysis. A maximum likelihood phylogeny was generated with IQ-TREE (v1.6.1) [21] using the best-fit model (GTR+F+ASC+R4) identified by ModelFinder [22]. BLASTN (using Nucleotide Collection (nr/nt) database) results indicate the BoNT subtype is B1.

### Sample preparation for proteomic analysis

Autoclaved cultures (10 mL) were centrifuged for 10 minutes at 5,000 x g to separate cell mass and supernatant (SN), which were processed separately downstream. Following centrifugation, SN was removed to a fresh tube and stored at 4°C until ready to be processed. To process the cell pellet, the pellet was washed once in 1 mL sterile phosphate buffered saline (PBS) and then resuspended in 0.5 mL 20% Trichloroacetic acid (TCA; in water). Cell pellet samples were vortexed and incubated for ~24 hours at -20°C. The following day, frozen samples were thawed and centrifuged to pellet precipitated cell material. Cell pellet samples were washed twice with ice cold acetone and any remaining acetone following washing was driven off by brief incubation at 60°C (5 minutes). TCA-precipitated cell pellets were resuspended in 100 μL lysis buffer (6M urea, 14.3mM β-mercaptoethanol in 50mM Tris-HCl pH8). Samples were heated 60 minutes at 60°C, then diluted with 900 μL 50mM ammonium bicarbonate in 0.1% TFA (trifluoroacetic acid) to reduce urea concentration <1M. Two microliters of Promega Trypsin gold was added to each sample to digest proteins, and samples were incubated overnight at 37°C with shaking. The next day, samples were removed and peptides were purified by C18 solid phase extraction (SPE; Phenomenex) according to the manufacturer protocol. Samples were spun down to pellet any solid debris that was remaining; the soluble fraction was processed. Cleaned peptide samples were brought to near dryness (Eppendorf Vacufuge Plus) and resuspended in 25 μL 0.1% TFA water. Finally, peptide concentration was determined by the bicinchoninic acid assay (BCA; Pierce) and samples were adjusted to 1 mg/ mL by diluting with additional 0.1% TFA for analysis.

Supernatant samples were processed separately from cell mass samples. SN samples (9 mL) saved at 4°C were transferred to 40 mL round-bottom autoclaved centrifuge tubes, and 1.8 mL 100% trichloroacetic acid was added to each sample (final concentration 20% TCA). SN samples were vortexed and incubated at -20°C overnight. The next day, samples were centrifuged (8,000 x g, 4°C for 10 minutes) and pellets were washed with 1 mL acetone (total of 3 washes). Excess acetone was driven off by a short (5 minute) incubation at 60°C. Pellets were resuspended in 500 μL PBS containing 2% SDS and incubated at 4°C overnight. The following day, samples were sonicated for 60 minutes to break apart remaining solids. SDS was then removed from the samples using detergent removal spin columns (Pierce; 2 mL size) according to the manufacturer protocol. Sample volume following detergent removal was reduced to 100 μL (Eppendorf Vacufuge Plus) and added to 1.5 mL tubes with pre-weighed urea (36mg). One microliter of 1.43M β-mercaptoethanol was added to each sample prior to incubation for 60 minutes at 60°C. Trypsin digestion, SPE clean-up, and sample preparation for MS analysis were performed exactly as described for cell mass samples above.

### LC-MS/MS analysis

Peptide samples were separated with an Agilent 1200 HPLC containing a 40 cm long 0.15 mm ID fused silica packed with Jupiter 5 μm C-18 resin. For all samples, 1 μg was analyzed (each at 1 μg/μL concentration). To establish the separation gradient, the relative concentrations of two solutions were varied: Solution A (5% acetonitrile, 0.1% formic acid in nano-pure water) and Solution B (95% acetonitrile, 0.1% formic acid in nano-pure water). The flow rate was 2 μL per minute with the reversed phase gradient transitioning from 0% solution B to 45% solution B over the course of 60 minutes. Following this step, wash and regeneration steps were performed. The eluate from the HPLC was directly ionized and transferred into the gas phase with an electrospray emitter (operated at 3.5 kV relative to the mass spectrometer interface). An Orbitrap XL system (Thermo Electron, Thousand Oaks, CA) was used with the ion transfer tube maintained at 200 °C and 200 V. An ion injection time was set for automatic gain control with a maximum injection time of 200 ms for 5x10^7^ charges in the linear ion trap. Dynamic parent ion selection was used, in which the top five most abundant ions were selected for MS/MS using a 3 *m/z* window. Each sample was analyzed three times by LC-MS/MS to generate technical triplicates.

### Data processing and analysis

Data were exported as Thermo RAW files and searched against matching databases via MS-GF+ [23]. Database searching was performed against matching genome sequences for all five strains (Table 1). All protein FASTA sequences were downloaded from PATRIC (http://www.patricbrc.org) or NCBI (https://www.ncbi.nlm.nih.gov/). Output from MS-GF+ was filtered to identify confident peptide spectrum matches using the SpecProb value. A SpecProb filter of < 1 x 10^−9^ was used, which yielded a false discovery rate between 0.05–0.08% in all cases. Spectral counts were calculated for each protein by summing the number of times all peptides from that protein were observed in each sample run. Spectral counts were used as a measure of relative protein abundance in this study. Further filtering for confident identifications was performed by removing proteins with spectral counts equal to one, and those that were observed in less than 10% of datasets for each strain/condition/cell fraction data matrix. Filter-passing protein identifications (S1 Table) are defined as passing both the SpecProb and observation count filtering criteria. Statistical analysis and visualization of data were performed in the programs Inferno (http://code.google.com/p/inferno4proteomics/) and MeV (http://mev.tm4.org). Protein expression data was statistically compared by t-test.

## Results

### Sample generation and statistical analysis of protein expression changes

One important goal of this study was to use proteomic analyses to better understand the biology of *C*. *botulinum*, as little complete proteomic analysis of this pathogen has been published to our knowledge. Thus, we sought to characterize the protein expression profiles of five Clostridial strains, including four toxin-producing *C*. *botulinum* strains and one closely related non-toxigenic *C*. *sporogenes* comparator strain (Table 1). The genome sequence of each strain was translated into protein sequences and used in search algorithms in this proteomic analysis. Four matching sequences were publicly available, and we sequenced *C*. *botulinum* ATCC 17841 in order to have appropriate genome/proteome sequences for these samples. *C*. *botulinum* ATCC 17841 was found to be a B1 subtype. Phylogenetic analysis of the strains used in this study was performed. The *C*. *botulinum* strains are closely related within the Group I species group, while the *C*. *sporogenes* strain is within a distinct clade that includes both BoNT-producing and non-neurotoxigenic members (Fig 1).

Each strain was cultured in two medium types and samples were collected at three time points during growth. Both cellular material and supernatant from each sample were analyzed by LC-MS/MS in triplicate. Cell mass and supernatant were separated for analysis to reduce sample complexity. In all, this studied yielded ~360 datasets total (5 strains x 3 time points x 2 media types x 2 (duplicate biological replicates cultured on separate days) x 3 (triplicate technical replicate analyses by LC-MS/MS) x 2 cellular fractions (SN and cell pellet) = 360). All filter-passing protein identifications for each strain have been provided in a supplemental table (S1 Table).

The total number of filter-passing proteins identified in all samples ranged from 549 to 633, depending on the strain (S2 Table). Of that total, some proteins were only identified in the cell pellet fraction, some were only identified in the SN fraction, and some were identified in both fractions. The large number of proteins observed in both fractions is not unexpected, and can be attributed to several potential factors: 1) proteins contained in the cell are naturally secreted during growth and/or released during cell lysis, 2) samples were autoclaved for biosafety reasons, which may have contributed to additional release of cellular factors into the surrounding media post-growth, and 3) centrifugation was used to separate cell material from SN during processing, which likely allows some mixing of proteins between fractions. As the reasoning for separating cell mass and SN fractions was to increase depth of proteome coverage (i.e. by reducing sample complexity), and not to make conclusions about protein localization within the cell/culture, this amount of protein “crossover” was not of concern. Because > 95% of the proteins observed were identified in the cell fraction, we chose to focus on data from cellular samples, except where noted otherwise in the text.

**Fig 2:**
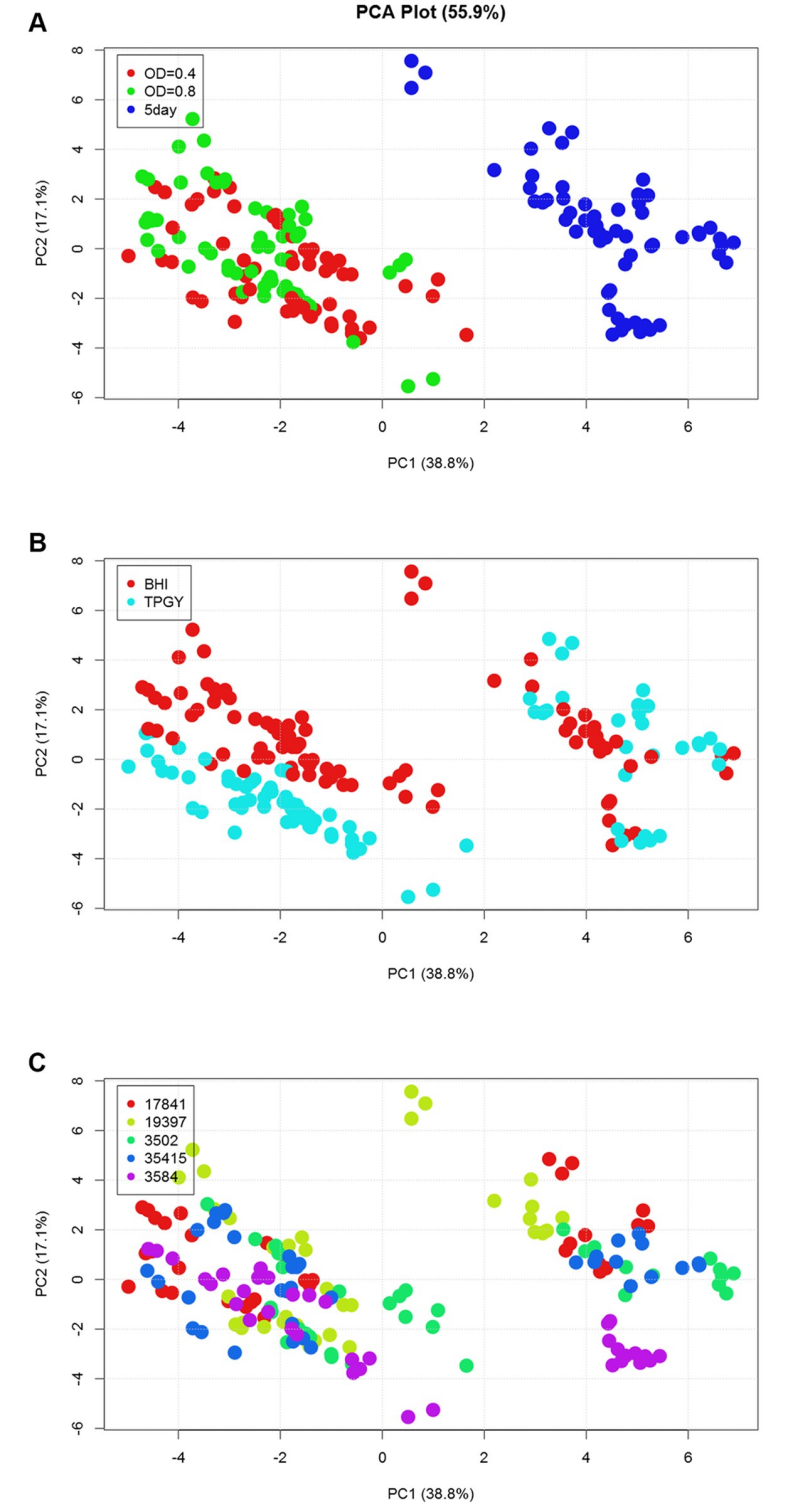
Principal component analysis reveals growth phase as a major factor in protein expression patterns. Cell mass protein expression data (post-filtering) from all strains, including that at all time points and in both medium types, was analyzed by PCA. (A) Separation on PC1 (x-axis) demonstrates that a major influence on protein expression patterns is the growth phase from which cells were harvested, as datasets from the early time points (OD_600_ = 0.4 and 0.8; red and green, respectively) segregate from those taken at the late time point (5 days; blue). While samples clearly separate into early and late growth phase “groups” in part (A), (B) medium type shows a slight differentiation in data (mostly from the early time points). A similar grouping is not observed when data are colored by (C) strain identity. The same plot is shown in parts A-C, and has been colored by different factors for visualization.

These data were analyzed to characterize the influence of growth phase, growth medium, and strain on protein expression. Protein expression was most significantly influenced by growth phase in all strains analyzed. All protein expression data was analyzed by principal component analysis (PCA). A plot of the data using the first two principal components (PCs) provides a visualization of overall relatedness of protein expression, showing clusters of samples that have similar proteomes. Expression profiles of early phase samples, including both OD_600_ = 0.4 and OD_600_ = 0.8 time points, were more closely related to other early phase samples, and expression profiles of late phase samples were more similar to those of other late phase samples, as indicated by their clustering closer together in the PCA plot (Fig 2A). Some clustering was observed based upon medium type (Fig 2B), although this separation was less distinct than clustering by growth phase. However, if colored according to strain identity (Fig 2C), there was no obvious delineation, suggesting that all five strains exhibit similar protein expression profiles.

Because growth phase was clearly an important factor in protein expression, we performed statistical analysis (t-test, see Materials and methods) to identify the subset of proteins that showed significantly different expression patterns between early (defined as data from two time points during exponential phase; OD_600_ = 0.4 and 0.8) and late (defined as data from the 5 day post-inoculation time point) growth phases. We also performed statistical analysis of protein expression in BHI vs. TPGY medium, although this effect was more subtle. All t-test results are presented in S3 Table. As detailed below, a significant switch in metabolic functions, as evidenced by expression of proteins in specific metabolic pathways, occurred during growth of *C*. *botulinum*.

### Protein expression of *C*. *botulinum* metabolic programs are reproducible and indicative of growth phase

**Fig 3:**
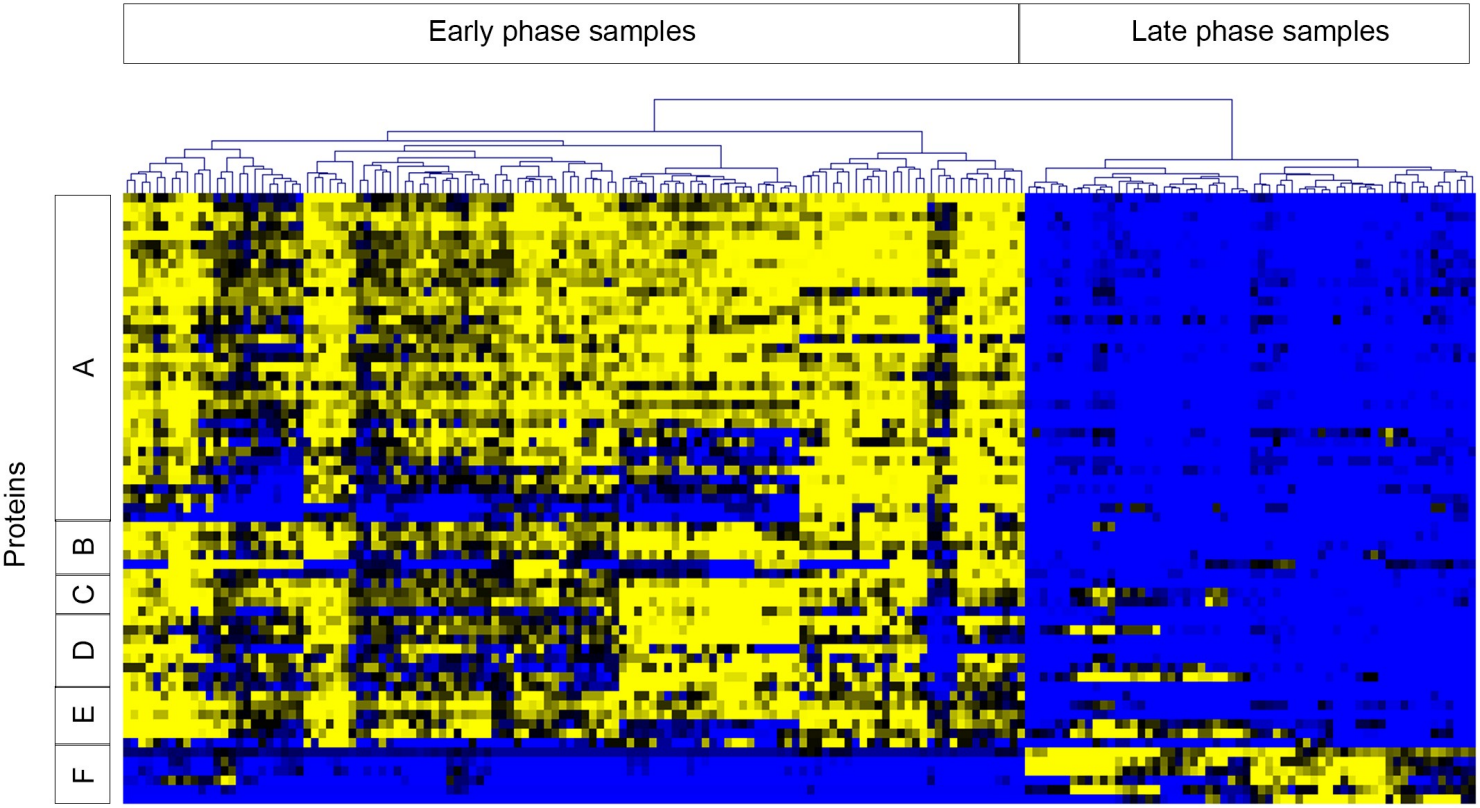
Proteins involved in growth and metabolic pathways are differentially expressed during early and late growth phases. Expression of proteins from many metabolic pathways/processes in early and late growth phases were significantly different by t-test. For visualization, proteins with significantly different expression profiles from six pathways/processes were selected: A) ribosomal proteins, B) translation/transcription elongation and initiation factors, C) RNA Polymerase subunits and sigma factor, D) tRNA synthetases, E) glycolytic enzymes, and F) enzymes of the glycine reductase system. Clusters A-E demonstrate higher expression during early time points and lower expression during late phase growth, while the reverse is true for cluster F. Data included in this analysis were z-score transformed spectral count values for selected proteins in cell fractions from all strains. Samples are represented across the horizontal axis and proteins along the vertical axis. Clustering of samples across the top separated early and late time point samples as noted. Heat map coloring is from blue (low expression) to yellow (high expression).

Growth in exponential phase enriched for expression of ribosomal proteins, glycolytic enzymes (i.e. triosephosphate isomerase, phosphofructokinase, fructose-1,6-bisphosphate aldolase, phosphoglycerate kinase, phosphoglyceromutase, and pyruvate kinase), enzymes involved in acetate and arginine fermentation, and proteins involved in DNA replication (DNA Polymerase, DNA gyrase, DNA topoisomerase), RNA transcription (RNA polymerase sigma factor, RNA polymerase subunits), and mRNA translation (elongation factors, tRNA synthetases). Conversely, stationary phase growth induced higher expression of the botulinum neurotoxin complex and sporulation-related proteins, as well as enzymes involved in butyrate fermentation, the glycine cleavage system, and the glycine reductase system. The expression patterns of proteins from a subset of these significantly changing metabolic pathways/processes are shown in Fig 3. These expression features are consistent with the idea of altered metabolic activity in the exponential and stationary phases of growth, and were observed reproducibly in both medium types and in all strains examined.

**Fig 4:**
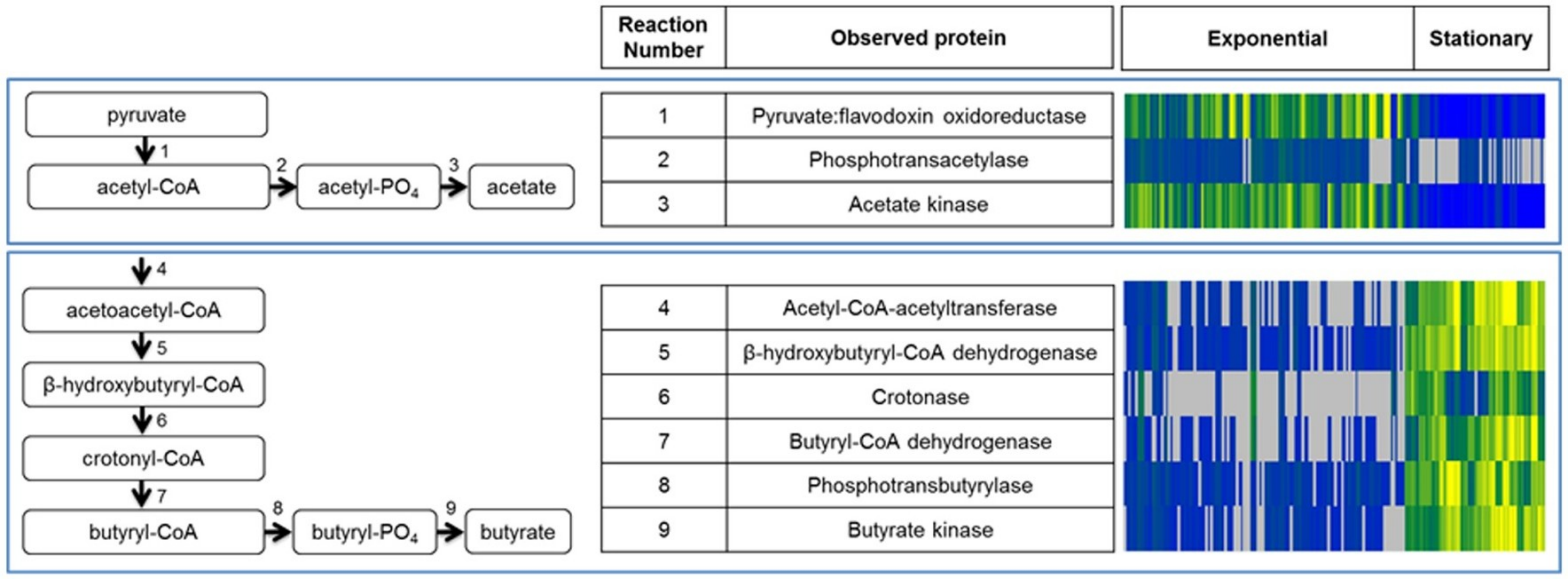
Growth phase-dependent expression of enzymes involved in acetate and butyrate fermentation. Proteins involved in the fermentation pathways shown were identified in this study, with enrichment of acetate fermentation (A) during exponential phase growth, and butyrate fermentation (B) during stationary phase growth. All enzymes in these pathways were identified across all strains and in both medium types examined, and expression patterns are shown in the heat map on the right hand side for each enzyme identified in the pathways. Blue shading indicates lower protein expression, yellow shading represents higher protein expression, and gray indicates that the protein was not observed in the sample. Protein expression data from all cell fraction samples are included in the heat map, shown from left to right as columns in the heat map.

The anaerobic metabolism of *Clostridia* relies upon fermentative pathways, such as the production of acetate and butyrate, and the breakdown of amino acids for energy [24]. Pyruvate and acetyl-CoA can feed into a fermentative pathway to produce acetate and butyrate (Fig 4). This study identified every enzyme in this pathway, with expression increased during exponential phase for the conversion of pyruvate to acetate (Fig 4A; pyruvate-ferridoxin oxidoreductase, phosphotransacetylase, and acetate kinase), and enriched during stationary phase for further conversion to butyrate (Fig 4B; thiolase, β-hydroxybutyryl-CoA dehydrogenase, crotonase, butyryl-CoA dehydrogenase, phosphotransbutyrylase, and butyrate kinase). This process is critical for ATP production in *Clostridia* via substrate level phosphorylation, and our data support the conservation of this mechanism across toxin-producing and non-toxigenic strains. These proteins were observed in all five strains examined, and were enriched during specific growth phases for both medium types.

**Fig 5:**
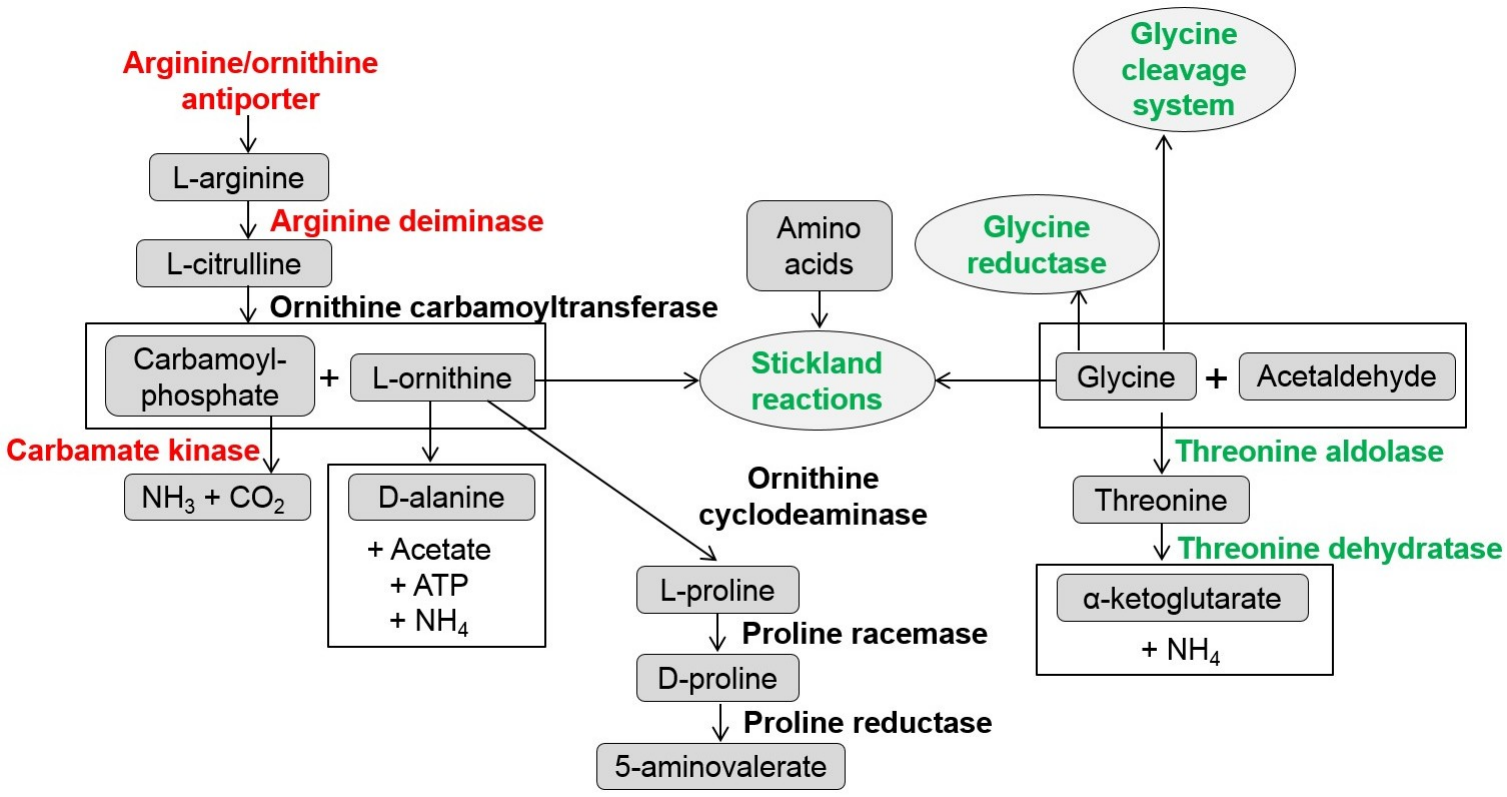
Metabolic pathways related to protein and amino acid metabolism. A subset of proteins were identified which are involved in the breakdown of proteins and amino acids for energy. This partial metabolic network shows how these metabolic features are influenced by growth phase. Red text indicates enriched observation of the protein during exponential phase, while green indicates enrichment at later stationary phase. Black indicates mixed observation during different growth phases.

Proteins (and/or their composite amino acids) are a significant energy source for *Clostridia*. A complex network containing multiple integrated metabolic pathways exists for the use of various amino acid substrates, leading to ATP generation via substrate level phosphorylation. Our study identified peptidases as well as proteins necessary for amino acid fermentation, and many of these proteins demonstrated growth phase dependent enrichment. Fig 5 presents a subset of reactions integral to protein breakdown that highlights proteins observed in this study. Central to this network and common in amino acid-rich conditions are Stickland reactions, which generate energy through paired oxidation and reduction reactions between two amino acids [25]. We observed proteomic evidence of Stickland and related reactions in *C*. *botulinum* and *C*. *sporogenes*. Proteins integral to arginine fermentation were enriched during exponential growth (Fig 5, red text; arginine deiminase, ornithine carbamoyltransferase). In stationary phase, proteins supporting the role of glycine as both an electron donor and electron acceptor was observed with the enriched expression of proteins of the glycine cleavage (glycerol kinase, glycine dehydrogenase subunits 1&2, glycine cleavage system aminomethyltransferase, and glycine cleavage system protein H) and glycine reductase (alpha, beta, and gamma components of the glycine reductase complex) systems (Fig 5; green text). The reproducible expression patterns of proteins integral to the metabolic strategies of *Clostridia*, such as carbohydrate and protein catabolic pathways, provide tangible targets for possible signatures of growth. Interestingly, we observed these trends in all strains analyzed (including the non-toxigenic *C*. *sporogenes*) and in both medium types, which further supports the consistent nature of protein expression.

### Expression of the botulinum neurotoxin complex in toxigenic strains

**Fig 6:**
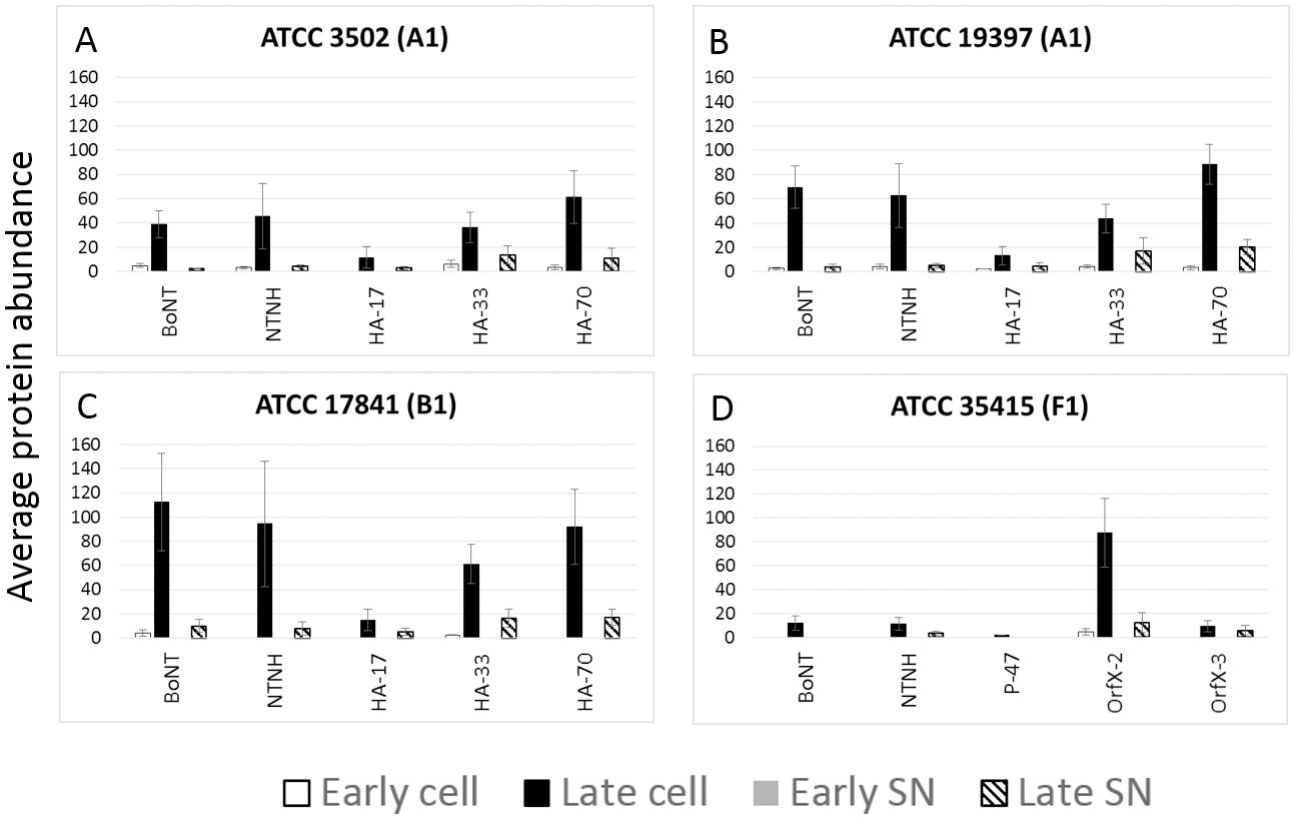
Botulinum neurotoxin and toxin-associated protein expression is highest in late phase growth. *C*. *botulinum* (A) ATCC 3502, (B) ATCC 19397, and (C) ATCC 17841 express botulinum neurotoxin, non-toxin non-hemagglutinin (NTNH), and three hemagglutinin proteins (HA-17, HA-34, and HA-70), while (D) ATCC 35415 expresses botulinum neurotoxin, NTNH, and a different complex of associated proteins, including P-47, OrfX-2, and OrfX-3. The additional OrfX-1 protein was not detected in our experiments. Average protein abundance (spectral counts) of toxin complex proteins in cell and SN fractions are presented. Error bars denote standard deviation. Early time points (including OD600 = 0.4 and 0.8) are shown in white bars for cell fraction and gray bars for SN (note that all values were zero for early SN samples). The late time point (5 days) is represented in black bars for the cellular fraction and striped pattern for the SN fraction.

Neurotoxin and associated toxin complex proteins were identified from samples of all *C*. *botulinum* toxigenic strains (Fig 6). Early growth phases contained less toxin and toxin complex proteins, while samples at stationary phase had greater detectable levels of these proteins [6]. One of two main groups of accessory proteins are typical in toxigenic strains, which are referred to as the HA type and the OrfX type [26]. Three strains were analyzed that contain the HA type toxin complex: two A1 strains (ATCC 3502, Fig 6A; ATCC 19397, Fig 6B) and one B1 strain (ATCC 17841, Fig 6C). These three strains expressed the three hemagglutinin proteins to varying levels (HA17, HA33, and HA70) along with the accessory NTNH (non-toxin non-hemagglutinin) protein. The serotype F strain (ATCC 35415) expresses a toxin complex that contains the toxin and NTNH with different accessory proteins: P-47, OrfX1, OrfX2, and OrfX3. Our analyses identified the toxin and NTNH, as well as OrfX2 and OrfX3 (Fig 6D). OrfX1 was not detected in our analyses, which was consistent with previous analyses in which OrfX1 could not be detected in a *C*. *botulinum* A2 crude toxin extract [27] and a *C*. *botulinum* F Langeland extracted toxin sample [13]. OrfX1 was likely expressed at levels that fell below our limit of detection, and P-47 was observed near the limit of detection in a subset of replicate samples. We observed neurotoxin and associated proteins in both cellular and SN fractions, although counts were higher in the cellular fractions. In some cases, expression was also detected in early cellular (but not SN) fractions. Altogether, expression of these proteins provides identifying information about toxin serotype.

### Developing internally normalized relative protein abundance signatures

A second goal of this study was to compare protein profiles of the strains that are reproducibly expressed in certain growth conditions, to determine if protein expression profiles could act as potential signatures for sample interrogation. Protein expression studies in a research laboratory setting are relatively easy to achieve, because a two-state comparison (such as treated/untreated) can be executed in a well-controlled manner. However, in some potential applications, such as characterization of a single unknown sample, a comparator sample would not be available. Therefore, a sample characterization strategy that includes internal normalization would be beneficial.

In this exploration of *Clostridium* protein expression, we identified a subset of proteins that were reproducibly enriched in samples during early or late phase growth. These proteins were involved in metabolic changes throughout growth, providing a subset of proteins that both make sense biologically and are highly likely to be observed in the sample (as they are integral to cell metabolism). We used the expression patterns of these proteins to test the hypothesis that relative protein abundances could be used in a simple way to deduce information about cultivation conditions. The spectral count measurements from two selected protein pairs (butyrate kinase and acetate kinase, and glycine reductase component B and 30S ribosomal subunit protein S13) were used to calculate a ratio of one protein to another within a single sample. These ratios were then averaged over all cell fraction samples (including all strains analyzed in this study) to generate the values shown in Table 2. The neurotoxin protein was purposely omitted because an unknown sample may not be derived from a toxigenic strain, and in addition other toxin detection methods exist. In these examples, the protein abundance ratios are less than 1 during early phase growth, and greater than 1 during late phase growth, correctly distinguishing growth phase. Although further testing of such protein signatures in additional strains and cultivation conditions, with multiple protein comparisons, would be needed to explore the reproducibility of protein expression across a wider breadth of samples, our study has provided the fundamental exploration of protein expression patterns in multiple strains and conditions, and has demonstrated the feasibility and usefulness of proteomic analysis in the characterization of unknown samples.

**Table 2:** Protein relative abundance ratios as signatures of cultivation conditions.

**Ratio butyrate kinase: acetate kinase**		
**ATCC strain number**	**Exponential**	**Stationary**
3502	0.20	6.93
19397	0.20	2.17
17841	0.11	3.65
35415	0.13	6.30
3584	0.15	12.40
**Ratio glycine reductase component B: 30S ribosomal subunit S13**		
**ATCC strain number**	**Exponential**	**Stationary**
3502	0.17	3.62
19397	0.18	6.64
17841	0.11	5.33
35415	0.14	7.70
3584	0.18	3.19

Relative protein abundances in cell fractions were used to calculate two proof-of-principle ratios to distinguish samples from early or late phase growth. Each ratio was calculated using two proteins that were identified in all samples and were consistently enriched in either exponential or stationary phase growth.

## Discussion

Protein expression profiles of five *Clostridium* strains, sampled from exponential and stationary growth phases and in two medium types, were characterized in this study. The Group I strains are known to be closely related from genetic comparisons, but DNA similarity could not predict protein expression profiles of these strains, including *C*. *sporogenes*. The analyses discussed herein provided the opportunity to examine the predicted proteins generated from genomic data in order to characterize the protein profiles observed in samples. While genomic information is critical for strain identification and classification, we sought to explore how proteome information may be used as a complementary method to reveal information about organism phenotype. Group I *C*. *botulinum* strains expressing different toxin types (BoNT/A, /B and /F) and non-toxigenic *C*. *sporogenes* exhibited similar protein expression profiles under the culture conditions examined in this study.

We explored three factors that may influence protein expression in these experiments: strain (and botulinum neurotoxin serotype of toxigenic strains), medium type, and growth phase. Growth phase from which samples were harvested was the most influential factor in protein expression. In all strains examined, the proteins most significantly altered between exponential and stationary phase, as determined by statistical analysis, were enriched in functions related to metabolic processes. Exponential phase growth enriched for expression of ribosomal proteins, acetate fermentation, and other growth-related functions such as transcription and translation. Conversely, growth in stationary phase was characterized by butyrate fermentation, amino acid metabolism, and neurotoxin expression in toxigenic strains. Toxin expression was observed with increased amounts detected at the late time point on day 5. It is unclear if maximum toxin expression may have occurred at a time that was prior to day 5, or if longer duration of culturing the strains may have revealed further increases in toxin expression among the three different toxins in these Group I strains. In addition to the toxin, the toxin cluster components (i.e. either HA proteins or the OrfX proteins) were detected with similar expression patterns as the toxin itself. Finally, sporulation is an important survival mechanism for *Clostridium* strains, and expression differences for specific sporulation proteins were subtle. This could be due to the short duration of the culturing or could have been the influence of culture conditions, such as media components, temperature, or specific strain characteristics.

The use of mass spectrometry and other protein-based assays to identify expressed toxin has been critically important in botulism diagnostics. We have used mass spectrometry to complement genetic information about five *Clostridium* strains by providing phenotypic information about strain protein expression during growth. In this study, we identified candidate protein signatures of cultivation conditions for *Clostridia* and demonstrated proof-of-principle examples of a straightforward ratio calculation method that uses relative protein abundance measurements to differentiate between samples. Although additional proteomic data from other strains, including those expressing other toxin types, those within other Group designations, and/or those grown under more distinct cultivation conditions (i.e. medium type or temperature) or prepared for proteomic analysis using different methods, will provide further understanding, this study generated the first proteome-level analysis of multiple *Clostridium* strains under varying growth conditions. The protein profiles identified herein, including but not limited to the toxin, offer a wealth of yet untapped information to assist in strain characterization for many applications.

## Supporting information

S1 Table Spectral count data for all filter-passing protein identifications. (XLSX)

S2 Table Number of filter-passing protein identifications by strain and sample type. (XLSX)

S3 Table Results of statistical analysis of effects of culture time point and culture medium type. (XLSX)
